# Down-regulation of sfrp1 in a mammary epithelial cell line promotes the development of a cd44^high^/cd24^low ^population which is invasive and resistant to anoikis

**DOI:** 10.1186/1475-2867-9-11

**Published:** 2009-05-07

**Authors:** Kelly J Gauger, Jeremy M Hugh, Melissa A Troester, Sallie Smith Schneider

**Affiliations:** 1 Pioneer Valley Life Sciences Institute, Baystate Medical Center, Springfield, MA 01199, USA; 2 Biology Department, University of Massachusetts, Amherst, MA 01003, USA; 3 Department of Epidemiology and Lineberger Comprehensive Cancer Center, University of North Carolina at Chapel Hill, Chapel Hill, NC 27599, USA; 4 Veterinary and Animal Sciences, University of Massachusetts, Amherst, MA 01003, USA

## Abstract

**Background:**

The Wnt family of secreted proteins is implicated in the regulation of cell fate during development, as well as in cell proliferation, morphology, and migration. Aberrant activation of the Wnt/β-catenin signaling pathway leads to the development of several human cancers, including breast cancer. Secreted frizzled-related protein 1 (SFRP1) antagonizes this pathway by competing with the Frizzled receptor for Wnt ligands resulting in an attenuation of the signal transduction cascade. Loss of SFRP1 expression is observed in breast cancer, along with several other cancers, and is associated with poor patient prognosis. However, it is not clear whether the loss of SFRP1 expression predisposes the mammary gland to tumorigenesis.

**Results:**

When SFRP1 is knocked down in a non-malignant immortalized mammary epithelial cell line (76 N TERT), nuclear levels of β-catenin rise and the Wnt pathway is stimulated. The SFRP1 knockdown cells exhibit increased expression of the pro-proliferative Cyclin D1 gene and increased cellular proliferation, undergo a partial epithelial-mesenchymal transition (EMT), are resistant to anchorage-independent cell death, exhibit increased migration, are significantly more invasive, and exhibit a CD24^low^/CD44^high ^cell surface marker expression pattern.

**Conclusion:**

Our study suggests that loss of SFRP1 allows non-malignant cells to acquire characteristics associated with breast cancer cells.

## Background

The most frequently occurring cancer in women, and the second leading cause of cancer death among women, is breast cancer. Cancer results from cellular mutations that enhance proliferation, decrease anti-proliferative signals, and decrease programmed cell death; and from cellular alterations that enhance angiogenesis and metastasis [1]. Members of the Wnt family of secreted proteins regulate cellular proliferation, morphology, and migration by way of β-catenin-mediated transcriptional activation [2-4]. Inappropriate activation of the Wnt/β-catenin pathway, which results from mutations in several downstream genes, contributes to the genesis of a wide range of human cancers [2]. However, such mutations are rarely observed in breast cancer despite the finding that β-catenin is stabilized in a majority of human breast tumors [5]. In addition, aberrantly activated Wnt signaling leads to inappropriate mammary gland development and mammary tumorigenesis in mice [3].

The Wnt family of secreted proteins is implicated in the regulation of cell fate during development, as well as in cell proliferation, morphology, and migration [2-4]. The best characterized Wnt pathway is the canonical Wnt/β-catenin pathway whereby Wnt signaling leads to the stabilization of β-catenin and activation of β-catenin-responsive gene expression. Wnt ligands activate the Wnt/β-catenin signaling pathway by binding to receptors comprised of Frizzled proteins in conjunction with one of the LDL receptor-related proteins LRP5 or LRP6. Receptor activation results in the ability of a cytoplasmic protein, Dishevelled (Dsh), to inhibit a multiprotein complex that includes APC, Axin, and the kinase GSK3β. Under normal circumstances, this complex is responsible for degrading the free cytosolic pool of β-catenin via phosphorylation of specific serine and threonine residues on the N-terminal, which ultimately targets the protein for ubiquitination and proteolysis. When this complex is dissociated as a result of Wnt signaling, β-catenin is less efficiently phosphorylated and ubiquitinated, which leads to increase nuclear β-catenin levels. In the nucleus, β-catenin forms a complex with the TCF/LEF1 family of HMG box transcription factors and stimulates the expression of specific target genes including the cyclin D1 oncogene.

Secreted frizzled-related proteins (SFRPs) are a family of Wnt antagonists which contain a cysteine-rich domain that is homologous to the Wnt-binding domain of frizzled receptor proteins [6]. However, SFRPs do not contain a transmembrane domain and therefore reside in the extracellular compartment where they antagonize Wnt signaling by binding to Wnt ligands and prevent ligand-receptor interactions and signal transduction [7]. Loss of SFRP expression is found in a multitude of cancers including breast cancer [8-10], cervical cancer [11], hepatocellular cancer [12], urothelial cancer [13], head and neck squamous cell cancer [14], lymphocytic leukemia [15], lung cancer [16], neuroectodermal cancer [17], bladder cancer [17], ovarian cancer [18], mesothelioma [19], endometrial cancer [20], and cervical cancer [11].

SFRP1 is a member of this protein family that is significantly downregulated in breast tumors and in breast carcinoma cell lines [8,9]. Moreover, loss of SFRP1 expression is associated with poor overall survival in patients with early breast cancer [10]. The loss of SFRP1 expression occurs both at the RNA level as demonstrated by *in situ *hybridization [21] and at the protein level as shown by immunohistochemistry [10]. Recent data have revealed that like many of the cancers described above, promoter hypermethylation is the reason for loss of SFRP1 mRNA and protein expression [22,23].

Currently, it is unknown whether SFRP1 loss causes the mammary gland to be predisposed to breast cancer development. This work was initiated to investigate whether loss of SFRP1 expression renders mammary epithelial cells susceptible to transformation *in vitro*. We demonstrate that when SFRP1 is downregulated in a non-malignant immortalized mammary epithelial cell line, sensitivity to Wnt signaling is enhanced and the cells exhibit distinct hallmarks of cancer progression.

## Results

### Characterization of 76 N TERT cells stably transfected with siSFRP1

**Figure 1:**
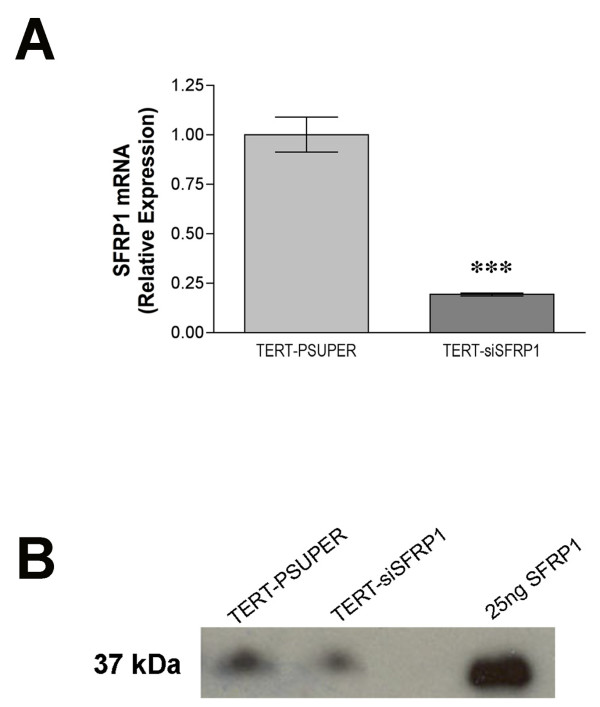
**The relative expression levels of SFRP1 mRNA and protein are reduced in TERT-siSFRP1 cells**. A, Total RNA was isolated from each cell line for real-time PCR analysis. The level of SFRP1 mRNA was normalized to amplification of GAPDH mRNA, which was performed in parallel wells for each cell line. Bars represent mean ± SEM SFRP1/GAPDH and are expressed as relative expression of TERT-PSUPER cells. ***p < 0.001 (significantly different from TERT-pSUPER cell line using student's *t*-test). B, The proteins from TERT-pSUPER and TERT-siSFRP1 supernatants were collected, concentrated, and SFRP1 expression was assessed by Western Blot Analysis. Twenty-five ng of recombinant SFRP1 was loaded onto the gel to confirm SFRP1 band size and specificity.

To evaluate the effects of SFRP1 down-regulation in an immortal mammary epithelial cell line, 76 N TERT cells were stably transfected with the pSUPER-siSFRP1 construct. To confirm that the expression level of SFRP1 was knocked down in siSFRP1 transfected cells, total RNA was isolated from vector transfected 76 N TERT cells (TERT-pSUPER) and siSFRP1 transfected cells (TERT-siSFRP1). Real-time PCR analysis revealed that the mRNA expression levels of SFRP1 are significantly lower in TERT-siSFRP1 cells when compared with TERT-pSUPER cells (Figure 1A). Additionally, to confirm that TERT-siSFRP1 cells secrete lower levels of SFRP1 protein, supernatants were collected from TERT-pSUPER and TERT-siSFRP1 cells and subjected to western blot analysis. Indeed, there is less SFRP1 protein in the media collected from TERT-siSFRP1 cells (Figure 1B).

### Evaluation of Wnt/β-catenin signaling in TERT-siSFRP1 cells

**Figure 2:**
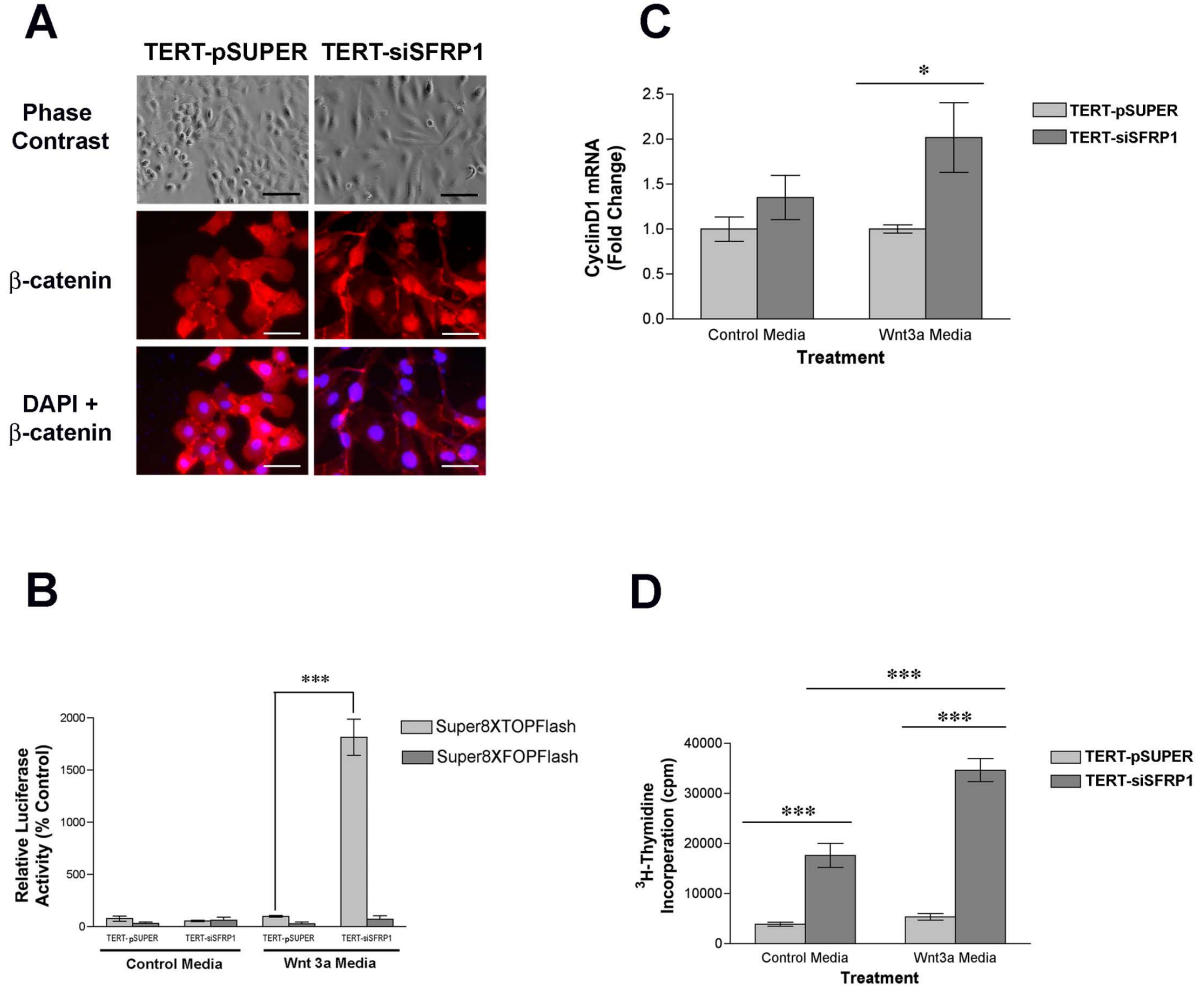
**Loss of SFRP1 results in an increase in Wnt-mediated β-catenin activity**. *A*, TERT-pSUPER and TERT-siSFRP1 cells were immunostained with anti-β-catenin and counterstained with DAPI phase contrast images were captured at 20× magnification (scale bar 100 μm) and fluorescent images were captured at 40× magnification (scale bar 50 μm). *B*, TERT-pSUPER and TERT-siSFRP1 cells were transfected with either the Super8XTOPflash or Super8XFOPflash vectors and relative luciferase activity was measured after an overnight incubation in the presence or absence of the Wnt3a ligand. Bars represent mean ± SEM of relative luciferase activity (firefly luciferase activity/renilla luciferase activity) normalized to the relative luciferase activity in TERT-pSUPER cells treated with control media. *C*, Twenty-four hours after the cells were treated with either control medium or Wnt3a medium, total RNA was isolated for real-time PCR analysis. The level of Cyclin D1 mRNA was normalized to amplification of GAPDH mRNA, which was performed in parallel wells for each treatment. *D*, Twenty-four hours after the cells were treated with ^3^H-Thymidine in either control media or Wnt3a media, the cells were lysed and total cpm was measured. Experiments were performed in at least triplicate wells and bars represent mean ± SEM cpm. *p < 0.05, ***p < 0.001 [significantly different from corresponding TERT-pSUPER cell line using Bonferoni's *t*-test following two-way ANOVA].

Considering that SFRP1 antagonizes the Wnt/β-catenin pathway, we next sought to determine whether reduced levels of SFRP1 would activate β-catenin signaling. Translocation of β-catenin from the cytoplasm to the nucleus is a strong indication that Wnt signaling is upregulated. TERT-pSUPER and TERT-siSFRP1 cells were grown on coverslips for 24 hours and phase contrast images illustrate the apparent morphology differences between the two cell lines (Figure 2A). The phenotypic changes observed when SFRP1 is knocked-down in 76 N TERT cells include a loss of cell polarity causing a spindle-cell morphology and an increase in the formation of pseudopodia. Moreover, fluorescent immunocytochemistry revealed that the morphological changes observed in TERT-siSFRP1 cells are accompanied by a marked decrease in cytoplasmic β-catenin protein expression levels along with a concomitant increase in nuclear β-catenin accumulation (Figure 2A). The nuclei were labeled with with 4', 6-diamidino-2-phenylidole to confirm that the cellular localization of β-catenin was indeed nuclear in TERT-siSFRP1 cells.

Given that β-catenin accumulates in the nucleus of TERT-siSFRP1 cells, we tested the hypothesis that there would be an increase in β-catenin-mediated transcription. TERT-pSUPER and TERT-siSFRP1 cells were grown in either control medium or Wnt3a medium and transfected with Super8XTOPflash (a β-catenin driven firefly luciferase reporter vector) or Super8XFOPflash (a firefly luciferase reporter vector that has a point mutation in the β-catenin response element rendering the construct unresponsive to β-catenin activity) [24]. Twenty-four hours after transfection, luciferase activity was measured and we found that TERT-siSFRP1 cells exhibit a significant increase in relative luciferase activity when grown in the presence of the Wnt3a ligand (Figure 2B; F = 97.58_(1,12)_, P < 0.0001).

CyclinD1 is a well characterized downstream gene of Wnt/β-catenin signaling that is upregulated in response to β-catenin transcriptional activation. Therefore, we sought to determine whether in the presence of Wnt3a stimulated β-catenin activation, Cyclin D1 expression would be higher in TERT-siSFRP1 cells when compared with TERT-pSUPER cells. Cells were seeded and allowed to attach overnight. The media was then changed to either control media or Wnt3a media and total RNA was isolated 24 hours after the treatment media was added. Real-time PCR studies confirmed that Cyclin D1 mRNA levels are significantly higher when TERT-siSFRP1 cells are grown in Wnt3a media (Figure 2C; F_(1,8) _= 8.16, P = 0.0213).

Considering that an increase in Cyclin D1 expression is associated with an increase in cellular proliferation, our goal was to establish whether there was an associated increase in proliferation in TERT-siSFRP1 cells when compared with TERT-pSUPER cells. Cells were seeded, allowed to attach overnight, and the following day both cell lines were treated with ^3^H-Thymidine in either control media or Wnt3a media. The cells were lysed 24 hours later and total cpm was measured. The data obtained from ^3^H-Thymidine assays confirmed that TERT-siSFRP1 cells proliferate significantly more rapidly (Figure 2D; F_(1,32) _= 20.62, P < 0.0001).

### Evaluation of EMT associated gene expression in TERT-siSFRP1 cells

**Figure 3:**
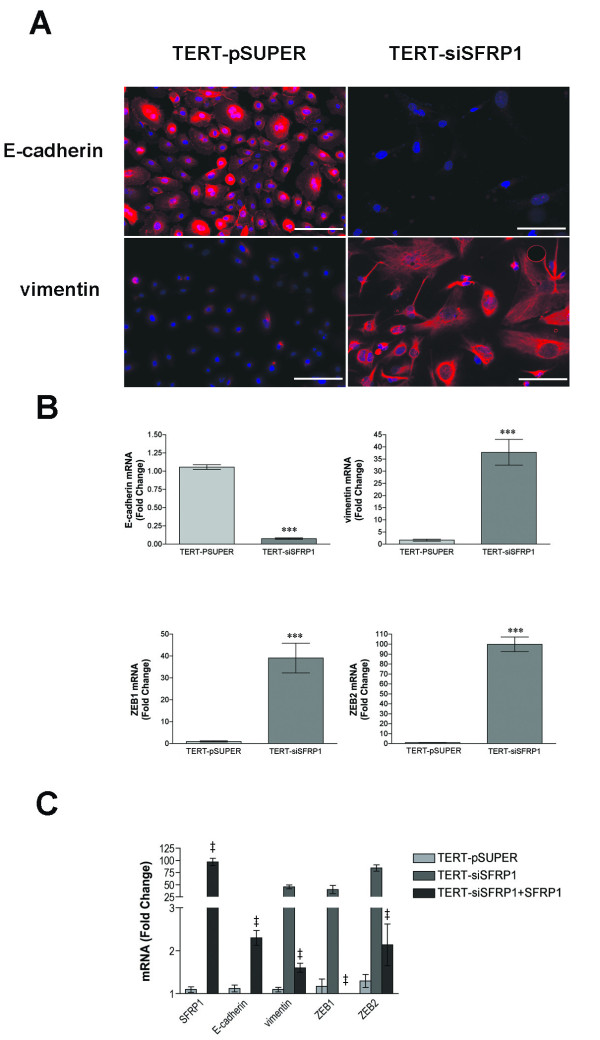
**TERT-siSFRP1 cells exhibit a gene expression profile that is consistent with partial EMT**. *A*, TERT-pSUPER and TERT-siSFRP1 were immunostained with either anti-E-cadherin or anti- vimentin and counterstained with DAPI. *B*, For each cell line, total RNA was isolated from three separate harvests for real-time PCR analysis of E-Cadherin, vimentin, ZEB1 and ZEB2. *C*, The SFRP1-pCDNA3.1 construct was transfected into TERT-siSFRP1 cells and RNA was isolated from 3 separate harvests. Real-time PCR analysis was performed to measure the relative expression of SFRP1, E-cadherin, vimentin, ZEB1 and ZEB2. All Real-time PCR results are from two separate experiments performed in triplicate and results were normalized to amplification of GAPDH mRNA. Bars represent mean ± SEM and are expressed as fold change with respect to TERT-pSUPER cells. *p < 0.05 ***p < 0.001 (significantly different from TERT-pSUPER cell line using student's *t*-test), ^‡^p < 0.001 (significantly different from TERT-siSFRP1 cell line using Bonferoni's *t*-test following one-way ANOVA). Immunostain images were captured at 40× magnification (scale bar 50 μm).

Epithelial-mesenchymal transitions (EMTs) are implicated in the conversion of early stage tumors into invasive malignancies. EMT is a process whereby epithelial cells lose polarity as well as cell-cell contacts and undergo a dramatic remodeling of the cytoskeleton resulting in a mesenchymal morphology. TERT-siSFRP1 cells appear to exhibit this phenotype exemplified by a switch from a polarized, epithelial phenotype to a more motile fibroblastoid phenotype. Down-regulation of E-cadherin, a central component of cell-cell adhesion junctions, is a hallmark of EMT. Both immunocytochemistry as well as real-time PCR analyses reveal that E-cadherin expression is significantly repressed in TERT-siSFRP1 cells (Figure 3A–B). In addition to the loss of E-cadherin expression, EMT is characterized by the acquisition of the mesenchymal marker vimentin. Vimentin is a type-III intermediate filament that is typically expressed in mesenchymal cells and it is only expressed in epithelial cells when they become involved in physiological or pathological processes requiring epithelial cell migration [25]. Immunocytochemistry and real-time PCR analyses demonstrate that vimentin is significantly upregulated in TERT-siSFRP1 cells (Figure 3A–B).

We next sought to determine whether key regulators of E-cadherin and vimentin expression are altered in TERT-siSFRP1 cells. Although the E-cadherin repressors Snail and Slug are modestly increased in TERT-siSFRP1 cells (data not shown), both ZEB1 (also known as TCF8 and δEF1) and ZEB2 (also known as ZFXH1B and SMAD-interacting protein 1 [SIP1]) are significantly increased in response to SFRP1 down-regulation (~40 fold and ~100 fold respectively, Figure 3B). These two E-box-binding transcription factors are important regulators in the complex network of transcriptional repressors that regulate the expression of E-cadherin and EMT through the repression of a number of master regulators of epithelial polarity [26-29].

Next, in order to confirm that observed EMT associated morphological changes in TERT-siSFRP1 cells are due to SFRP1 loss and not due to off-target siRNA effects, SFRP1 was re-expressed in these cells. Real-time PCR analysis was utilized to confirm that SFRP1 expression is up-regulated in TERT-siSFRP1 cells (TERT-siSFRP1+SFRP1). In addition, we are able to show that the expression of E-cadherin is elevated and that the levels of vimentin, ZEB1, and ZEB2 are reduced in TERT-siSFRP1+SFRP1 cells when compared with TERT-siSFRP1 cells indicating that they are direct SFRP1 targets (Figure 3C).

### Evaluation of the cellular characteristics exhibited by TERT-siSFRP1 cells

**Figure 4:**
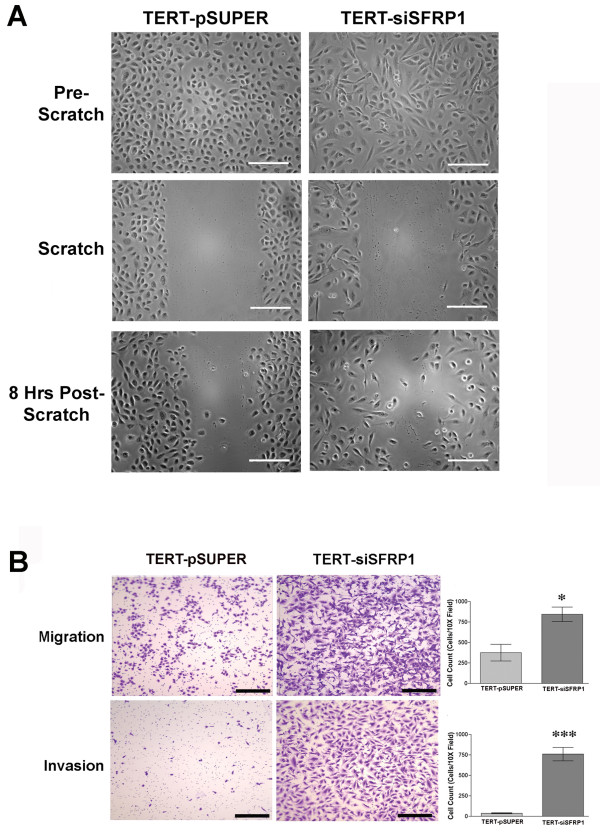
**Loss of SFRP1 increases the migratory and invasive behavior of 76 N TERT cells**. *A*, TERT-pSUPER and TERT-siSFRP1 cells were plated in 30 mm dishes and allowed to reach 100% confluence. A pipette tip was utilized to generate a wound (scratch) down the center of the plate. Image of the cells capable of migrating across the scratch 8 hours after the wound was created. Images were captured at 20× magnification; scale bar 100 μm. *B*, TERT-pSUPER and TERT-siSFRP1 cells that were plated in BD BioCoat™ control inserts or Matrigel™ invasion chambers and the cells capable of migrating through the 8 μm pore or invading the reconstituted basement membrane (Matrigel™) through the 8 μm pore towards a chemoattractant were stained with 10% crystal violet and counted. Images were captured at 10× magnification; scale bar 500 μm. (Experiments were repeated three times and the number of cells within a representative 10× field from each experiment were counted and bars represent mean ± SEM cell number. *p < 0.05,***p < 0.001 (significantly different from TERT-pSUPER cells using a student's *t*-test).

Considering that cells that have undergone EMT are more likely to metastasize, we next sought to measure the migratory and invasive properties of TERT-siSFRP1 cells. First, a simple scratch wound assay revealed that after just 8 hours, TERT-siSFRP1 cells are more motile then TERT-pSUPER cells (Figure 4A). Next, the cells were plated in BD BioCoat™ control chambers or Matrigel™ Invasion Chambers (BD Biosciences) and the cells capable of migrating through the 8 μm pore (migration) or through Matrigel™ (invasion) towards a chemoattractant were stained with 10% crystal violet and quantified. We clearly show that TERT-siSFRP1 cells are significantly more migratory and invasive then TERT-pSUPER cells (Figure 4B).

**Figure 5:**
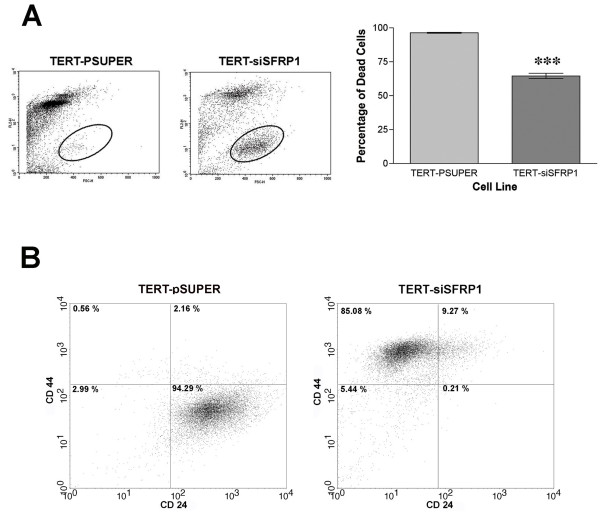
**TERT-siSFRP1 cells are anoikis resistant and exhibit CD44^high^/CD24^low ^cell surface marker expression pattern**. *A*, Cell culture dishes were coated with 1% agarose/DMEM, which was allowed to polymerize creating a barrier that prevented cellular attachment. TERT-pSUPER and TERT-siSFRP1 cells were plated in the wells and after 24 hours, FACs analysis was performed to compare the percentage of propidium iodide positive (FL2; dead cells) between cell lines. Scatter plot depicting FL2 positive cells vs. forward scatter (FSC-H) and oval represents area of viable cell not included in the analysis. Two separate experiments were performed in triplicate and bars represent mean ± SEM percent cell death. ***p < 0.001 (significantly different from control TERT-pSUPER cells using student's *t*-test) B, TERT-pSUPER and TERT-siSFRP1 cells were incubated with antibodies against human CD44-FITC and CD24-PE and labeled cells were analyzed by flow cytometry. FACS analysis reveals that TERT-pSUPER cells have a CD44^low^/CD24^high ^phenotype and TERT-siSFRP1 cells exhibit a CD44^high^/CD24^low ^phenotype.

Normal adherent cells are strongly dependent on adhesion to extracellular matrix for cell proliferation and undergo apoptosis if they are detached from the substratum, in a process known as anoikis [30]. In contrast, tumor cells can survive and grow without adhesion to a substratum; this is a critical step in tumorigenic transformation [30,31]. Cells were grown in anchorage-independent conditions and flow cytometry analysis showed that TERT-siSFRP1 cells are significantly more resistant to anoikis (Figure 5A).

Human metastatic breast cancer cell lines have a higher percentage of CD44^high^/CD24^low ^cells than less aggressive breast cancer cells lines [32]. Moreover, Mani *et. al*. recently showed that non-malignant mammary epithelial cells that have been induced to undergo EMT exhibit a CD44^high^/CD24^low ^cell surface marker expression pattern [33]. Figure 5B illustrates that TERT-siSFRP1 exhibit a striking population shift to a CD44^high^/CD24^low ^phenotype compared with TERT-pSUPER cells (which exhibit a CD44^low^/CD24^high ^phenotype). Interestingly, this CD44^high^/CD24^low ^cell surface maker expression pattern is associated with both human breast cancer stem cells and normal mammary epithelial progenitor cells [34].

### Genetic profile of TERT-siSFRP1 cells

To determine how SFRP1 loss affects additional different cellular processes, we performed a microarray analysis of TERT-siSFRP1 cells compared with TERT-pSUPER cells. One class SAM analysis identified 130 genes that were differentially regulated in SFRP1 knockdown cells (FDR = 4.7%). These genes, along with their average fold change relative to the parent are listed in Additional file 1. Ingenuity pathway analysis indicated that regulation of Cell Death (p = 3.99e-4 to 4.99e-2) was an important molecular and cellular function, with the following pro-survival genes being upregulated: MDM2, TMSB4X, ATRX, CCL25, and APH1A. These same genes play an important role in Cell-to-Cell Signaling and Interaction (p = 1.03e-3 = 4.33e-2) and Cellular Assembly and Organization (p = 1.46e-3 – 4.75e-2), and these categories were also significant in Ingenuity Pathway Analyses. MDM2 is an E3 ligase that is responsible for the ubiquination and subsequent degradation of the p53 tumor suppressor gene [35]. TMSB4X encodes an actin sequestering protein which is involved in angiogenesis, cell proliferation, migration, and differentiation [36,37]. The protein encoded by ATRX gene contains an ATPase/helicase domain and belongs to the SWI/SNF family of chromatin remodeling proteins. Mutations in this gene lead to mental retardation which may be explained by the finding that ATRX is involved in neuronal cell survival [38]. The cytokine encoded by CCL25 has chemotactic activity which has been shown to enhance the migration of several cell types including mesenchymal stem cells [39]. Finally, APH1A is a multipass transmembrane protein that interacts with presenilin and nicastrin as a functional component of the gamma-secretase complex which is required for the intramembrane proteolysis of Notch [40] and activation of this signaling pathway is associated with mammary oncogenesis [41].

## Discussion

The present study indicates that a reduction in SFRP1 expression allows 76 N TERT cells to acquire many of the properties observed in breast cancer cells. These SFRP1 knocked down cells (TERT-siSFRP1) have increased levels of β-catenin in their nucleus, which is indicative of β-catenin stabilization and activated Wnt/β-catenin signaling. Moreover, TERT-siSFRP1 cells undergo a dramatic phenotypic change which can be explained in part by a genotypic change that is consistent with EMT. Similar to malignant mammary cells, TERT-siSFRP1 cells are more resistant to anchorage-independent cell death, are quite migratory and invasive, and exhibit a CD44^high^/CD24^low ^cell surface marker expression pattern. Finally, microarray analysis revealed that the characteristics exhibited by TERT-siSFRP1 are consistent with affected pathways including cell death, cell-to-cell signaling, and interaction cellular assembly.

The downstream effector of Wnt signaling, β-catenin, exists in three different subcellular forms: membrane-bound (complexed with E-cadherin), cytosolic, and nuclear [42]. The data presented here show that β-catenin accumulates in the nucleus of TERT-siSFRP1 cells where it is available to act as a transcription factor. Considering that genes regulated by β-catenin are involved in tumorigenesis, the nuclear accumulation of β-catenin may be a possible mechanism by which SFRP1 renders the mammary gland more susceptible to tumorigenesis. Interestingly, Nakopoulou *et. al*. have demonstrated that increased nuclear staining for phospho-β-catenin in breast tumor specimens is associated with poorer overall survival (OS)[43]. Similarly, Veeck *et. al*. have found increased promoter hypermethylation of SFRP1 is also associated with poor prognosis [23]. Putatively, SFRP1 loss may be associated with nuclear β-catenin accumulation in human breast tumors. Interestingly, β-catenin-mediated transcription from an exogenous reporter construct was not activated unless exogenous Wnt3a was added to the media. These findings suggest that either greater amounts of nuclear β-catenin are necessary to see significant differences in reporter activity or that SFRP1 loss is affecting alternate pathways through either non-canonical Wnt signaling or Wnt-independent mechanisms.

Previously, Shulewitz *et. al*. ultilized siRNA oligonucleotides to transiently silence SFRP1 MCF-10A breast epithelial cells. In order to evaluate the permanent changes in cellular behavior that occur in response to SFRP1 down-regulation, we chose to create the TERT-siSFRP1 stable cell line to [44]. Our results are consistent with Shulewitz *et. al*. in that TERT-siSFRP1 cells exhibit an increase in β-catenin-mediated luciferase activity and CyclinD1 expression in response to Wnt3a stimulation. CyclinD1 is a cell-cycle regulator essential for G1 phase progression [45] and the pro-proliferative nature of this oncogene is implicated in pathogenesis of several human tumor types, including breast carcinomas [46]. Our findings clearly demonstrate that the increase in Cyclin D1 expression, due to aberrant β-catenin transcriptional activation, observed in TERT-siSFRP1 cells contributes to an increase in cellular proliferation. However, other proliferative cues must be affected by SFRP1 loss because the enhanced cellular proliferation occurs both in the presence as well as the absence of the Wnt3a ligand whereas Cyclin D1 expression is increased only when Wnt3a is added to TERT-siSFRP1 cells.

Tumor cell metastasis occurs when primary epithelial cells exit their site of origin and colonize at a distant site. In order for this process to occur, the cells must invade the extracellular matrix, migrate into blood vessels, and invade secondary organs. Here we show that when SFRP1 is down-regulated, 76 N TERT cells become significantly more migratory as well as invasive. The phenotypic changes observed when SFRP1 is down-regulated include a transformation from an epithelial cell morphology to a more fibroblast-like cell with reduced intercellular adhesion and increased motility. Interestingly, these characteristics define the EMT process, which is known to occur during tumor progression as well as metastasis. After careful assessment of several genes implicated in EMT, we can conclude that loss of SFRP1 expression likely pushes 76 N TERT cells into this pathophysiological transformation.

E-cadherin down-regulation is frequently associated with Wnt signaling and we show here that E-cadherin is significantly repressed in TERT-siSFRP1 cells. Loss of E-cadherin expression is emerging as one of the most common indicators of EMT onset, as it normally acts as a tumor suppressor by inhibiting invasion [47,48]. Considering that both ZEB1 and ZEB2 are key transcriptional repressors of E-cadherin, and the expression levels of ZEB1 and ZEB2 are drastically enhanced in response to SFRP1 down-regulation, it is reasonable to suspect that the loss in E-cadherin expression is a direct result of the increase in these transcription factors. The expression of vimentin is also frequently associated with the EMT process and is significantly upregulated in TERT-siSFRP1 cells. The increase in vimentin expression may be due to the ~100-fold increase in ZEB2 expression, as ZEB2 is also involved in up-regulating the expression of vimentin in breast cancer cells [49]. Interestingly, Gilles *et. al*. have shown that β-catenin can transactivate vimentin expression which could explain, in part, the increase in vimentin expression when SFRP1 is down-regulated [50].

It is well established that de-regulated expression and altered function of the genes involved in cell-cycle regulation and death contribute to the pathogenesis of cancer [51] and SFRP1 exhibits an apoptotic function in several different tissues, including breast epithelial cells [52-54]. However, the data presented here are the first to show that SFRP1 down regulation renders mammary epithelial cells resistant to cell death. Moreover, this characteristic is observed in anchorage-independent growth conditions and in the absence of exogenous Wnt3A ligand. Interestingly, caspase-1 is significantly down-regulated in TERT-siSFRP1 cells (data not shown). Although the role of caspase-1 down regulation has not been studied in breast cancer cells, it is lost in other cancer cell lines [55,56]. The microarray data provide some other suggestions as to how TERT-siSFRP1 cells become resistant to anoikis, such as the increase in the pro-survival gene, Mdm-2, which is a potent regulator of p53. We also noted an increase in ATRX, a member of the SWI/SNF family of chromatin remodeling proteins, which has been previously shown to be involved in neuronal cell survival [38]. However, further work is required to fully elucidate the mechanisms by which SFRP1 loss increases anchorage independent cell survival.

Metastatic breast cancer cells are often characterized by a CD44^high^/CD24^low ^cell surface marker expression pattern [32]. Although it is logical that cells that have undergone EMT and exhibit this cell surface marker expression pattern are able to break out from the primary tumor and metastasize to distant locations, it has remained poorly understood as to how such cells could self-renew to form secondary metastases. Recent work by Mani *et al*. has addressed this dilemma by genetically modifying non-malignant mammary epithelial cells to undergo EMT [33]. Not surprisingly, these cells were shown to exhibit a CD44^high^/CD24^low ^phenotype. Although this cell surface marker expression pattern is associated with metastatic potential, it is also observed in both normal as well as breast cancer stem cells [34]. Forced EMT allowed the cells to exhibit additional characteristics associated with progenitor cells including an increased ability to form primary as well as secondary mammospheres, differentiate into secondary structures, and express both luminal and basal/myoepithelial markers, which exemplifies the bipotency of these cells [33]. We have observed that loss of SFRP1 also results in primary and secondary mammosphere formation (data not shown) and we are currently investigating whether these cells have additional stem cell-like characteristics.

It is noteworthy to point out that non-conical Wnt signaling is also known to induce cell migratory phenotypes during developmentand isoften separated into two subcategories including the Planar Cell Polarity (PCP) pathway and the Wnt/Ca^2+ ^pathway [57]. The EMT associated phenotype observed in TERT-siSFRP1 cells may be explained in part by particular sections of the signal transduction cascades that characterize these pathways. First, PCP signals through Rho and Rac GTPases to alter cell polarization and affect the actin cytoskeleton resulting in an increase in cellular motility and directional migration. Second, the Wnt/Ca^2+ ^pathway can activate the GTPase CDC42, which forms a complex with Par3 and atypical protein kinase, and this pathway has been previously implicated in ErbB2 mediated EMT in mammary epithelial cells [58]. Finally, the up-regulation of TMSB4X, filamin beta, and ADAMTS15 noted in the microarray analysis support the pro-migratory and invasive features and hint at the possible role of either other non-canonical Wnt pathways and/or Wnt-independent EMT mechanisms also being induced by SFRP1 loss [59-61].

The reduction in SFRP1 could potentially alter other pathways, independent of Wnt signaling, which could contribute to a malignant transformation and progenitor cell phenotype. For instance, Notch signaling may very well be aberrantly activated in TERT-siSFRP1 cells and exacerbate their aggressive phenotype. Ayyanan *et. al*. previously showed that Wnt-1 treated HMECs undergo a malignant transformation which is a result of not only activated β-catenin, but also activated Notch signaling [62,63]. Our data lends support to the hypothesis that the loss of SFRP1 may facilitate Notch signaling. Specifically, TERT-siSFRP1 cells have increased levels of the APH1A gene, which is a member of the gamma-secretase complex required for the intramembrane proteolysis of and activation of Notch. Furthermore, Mdm-2 is an ubiquitin ligase which targets the Notch inhibitor, Numb, for degradation [64]. Lastly, Notch is frequently activated in CD44^hi^/CD24^lo ^breast epithelial cells and inhibition of gamma-secretase activity has been shown to block the ability of the cells to form mammospheres and sensitizes the cancer cells to undergo growth arrest and apoptosis [65,66]. Research is ongoing to determine the importance of Notch signaling in the physiological characteristics imparted by SFRP1 loss.

## Conclusion

The cell line that we have constructed (TERT-siSFRP1) has allowed us to look at the effects of reduced SFRP1 expression levels on subcellular localization of β-catenin as well cellular morphology, characteristics, behaviors, and genetic profile. This cell line will allow us to pursue future studies designed to assess the role of SFRP1 loss in tumorigenesis. Based on the available information and our findings, we propose that the maintenance of SFRP1 expression is especially important in preventing aberrant Wnt signaling and inappropriate cell behavior in the mammary gland. The finding that SFRP1 down-regulation promotes anoikis resistance, migration as well as invasion, and increases the population of CD44^High^/CD24^low ^is quite significant because it may partially explain why breast tumors with lost SFRP1 expression are associated with poor patient prognosis.

## Methods

### Plasmids and Constructs

The SFRP1 siRNA and plasmid was designed to generate the following RNA target sequence 5'-CUACGUGAGCUUCCAGUCG-3' [44]. The siSFRP oligonucleotide sequences: 5'-GATCCCCCTACGTGAGCTTCCAGTCGTTCAAGAGACGACTGGAAGCTCACGTAGTTTTTA-3' and 5'-AGCTTAAAAA CTACGTGAGCTTCCAGTCGTCTCTTGAA CGACTGGAAGCTCACGTAGGGG-3' were obtained from Integrated DNA Technologies (IDT; Coralville, IA) and annealed in 500 μM RNase-Free Duplex Buffer (IDT) and heated to 94°C for 2 min. The annealed oligonucleotides were compatible with and cloned directly into the BglII and HindIII sites of the pSUPER.retro vector (OligoEngine, Seattle, WA). The SFRP1-pCDNA3.1 construct was supplied by Dr. Yoshitaka Sekido (Aichi Cancer Center Research Institute, Nagoya, Japan). Both Super8xTOPFlash and Super8XFOPflash luciferase vectors were kindly provided by Dr. Randall Moon (University of Washington, Seattle, WA) and pRL-CMV was purchased from Promega (Madison, WI).

### Cell Culture

76 N TERT cells were obtained from Dr. Vimla Band and were routinely cultivated at 37°C in 5% CO_2 _and maintained in DMEM/F12 (GIBCO, Grand Island, NY) and the following components from GIBCO: 1% FBS, 1× Antibiotic-Antimycotic (100×), and 20 μg/mL Gentamycin. The following components from Sigma (St Louis, MO) were also used: 50 μM L(+)-Ascorbic acid sodium salt, 1 ng/ml Cholera Toxin Vibrio, 12.5 ng/ml Epidermal Growth Factor murine submaxillary, 2 nM β-Estradiol, 0.1 mM Ethanolamine, 1 μg/ml Hydrocortisone-Water Soluble, 1 μg/ml human Insulin solution, 0.1 mM O-Phosphorylethanolamine, 35 μg/ml bovine pituitary extract, 15 nM Sodium selenite, 10 μg/ml human apo-Transferrin, and 10 nM 3,3',5-Triiodo-L-thyronine sodium salt. 76 N TERT cells were plated at a density of 1.5 × 10^6 ^cells per 10 cm dish and transfected with 24 μg siSFRP1-PSUPER.retro using Lipofectamine™2000 (Invitrogen, Carlsbad, CA). The pSUPER.retro vector was trasfected into 76 N TERT cells to provide a negative control because the experiments use stably transfected populations, which were obtained by selection with 2 μg/ml puromycin (Sigma). To genereate TERTsiSFRP1+SFRP1 cells, TERT-siSFRP1 cells were grown in triplicate (30 mm plates; 5 × 10^5 ^cells/well) and 4 μg of the SFRP1-pCDNA3.1 vector was transiently transfected into the cells. Mouse fibroblasts that overexpress the Wnt3a ligand (ATTC# CRL-2647) and control mouse fibroblasts (ATTC# CRL-2648) were maintained at 37°C in 5% CO_2 _and cultured in DMEM supplemented with 10% FBS and 20 μg/mL Gentamycin (GIBCO). To generate conditioned media, both mouse fibroblast cell lines were split 1:10 and allowed to grow for 4 days (approximately to confluency). The media was removed and sterilized by passing through a 0.2 μm filter. 10 ml of fresh culture media was be added to the cells which were cultured for another 3 days. The media was removed, sterilized by passing through a 0.2 μm filter, and mixed with the first batch of media 1:1.

### RNA Isolation and Real-Time PCR

Total RNA was extracted from cells using an acid-phenol extraction procedure [67], according to the manufacturer's instructions (Trizol, Invitrogen, Carlsbad, CA). Relative levels of mRNA were determined by quantitative real-time PCR using the Mx4000™ real time PCR system (Stratagene, La Jolla, CA) and all values were normalized to the amplification of GAPDH. The PCR primer sequences are listed in Additional file 2. The assays were performed using the 1-Step Brilliant^® ^SYBR^® ^Green QRT-PCR Master Mix Kit (Stratagene) containing 200 nM forward primer, 200 nM reverse primer, and 100 ng total RNA. The conditions for cDNA synthesis and target mRNA amplification were performed as follows: 1 cycle of 50°C for 30 min; 1 cycle of 95°C for 10 min; and 35 cycles each of 95°C for 30 s, 55°C for 1 min, and 72°C for 30 s.

### Western Blot Analysis

Following 1 week in culture, the supernatant of TERT-pSUPER and TERT-SFRP1 cells was collected and concentrated using the Nanosep 10 K Omega kit (Pall Corporation, Ann Arbor, MI) according to the manufacturer's instructions. Protein concentration was quantified using the BCA™ Protein Assay Kit (Pierce, Rockford, IL). A total of 100 μg of protein was run on a 10% SDS-Page gel and transferred to a PVDF membrane. The membrane was blocked for 45 minutes with 5% milk in tris-buffered saline containing 0.05% Tween-20 (TBS-T). The primary antibody [rabbit anti-human SFRP1 (Rockland, Gilbertsville, PA)] was diluted 1:200 and incubated overnight at 4°C. The secondary antibody [goat anti-rabbit IgG-HRP (Santa Cruz Biotechnology, Santa Cruz, CA)] was applied (1:5000) and incubated for 45 minutes at room temperature. The blot was washed and developed using a Western Blot Luminol Reagent (Santa Cruz Biotechnology).

### Fluorescent Immunocytochemistry

TERT-pSUPER and TERT-siSFRP1 cells (5 × 10^4 ^cells/well) were plated on glass coverslips in a 24 well plate and allowed to adhere overnight. The following day, the media was removed, cells were rinsed with 1× PBS, and fixed in 3.7% formaldehyde for 10 min at room temperature. Next cells were permeabilized with 0.5% TritonX-100 for min at 4°C followed by 3 15 min washes with PBS:glycine (130 mM NaCl, 7 mM Na_2_HPO_4_, 3.5 mM NaH_2 _HPO_4_, 100 mM glycine). Cells were then blocked in IF buffer (130 mM NaCl, 7 mM Na_2_HPO_4_, 3.5 mM NaH_2 _HPO_4_, 7.7 mM NaN, 0.1% BSA, 0.2% TritonX-100, 0.05% Tween 10) plus 10% goat serum (GS) for 1–2 hrs and subsequently with 2° blocking buffer [IF buffer containing 10% GS and 20 μg/ml goat anti-mouse F(ab')_2_] for 30–45 min. Rabbit anti-β-catenin (Millipore, Billerica, MA) was diluted 1:500, mouse anti-E-cadherin (BD Transduction, Mississauga, ON. Canada) was diluted 1:50, and mouse anti-vimentin (clone 9; Theremoscientific, Waltham, MA) was diluteld 1:50 in 2° blocking buffer and incubated overnight at 4°C. Unbound 1° antibody was removed by washing 3× in IF buffer for 15 min each and then an anti-rabbit or anti-mouse 2° antibody coupled with Alexa Fuor-568 (Molecular Probes, Invitrogen) was diluted 1:500 in IF buffer containing 10% GS and incubated for 45–60 min. Unbound 2° antibody was washed as described above. The coverslips were removed from the wells, mounted onto microscope slides with Vectashield Mounding Medium for Fluorescence with DAPI (Vector Laboratories Inc, Burlingame, CA), and images were captured with a Nikon Eclipse TE2000-U at the same exposure and gain using Metaview™ software (Universal Imaging Corporation, Downingtown, PA).

### Transient Transfection and Luciferase Assay

A total of 1 × 10^5 ^TERT-pSUPER or TERT-siSFRP1 cells/well were plated in a 24 well plates and were transfected the following day with 0.8 μg of either Super8xTOPFlash or Super8XFOPflash as well as 0.08 μg pRL-CMV. After a 6 hour incubation, the media was removed and replaced with either control medium or Wnt3a medium. Twenty-four hour after media treatment, cells were washed with 1× PBS and lysed using passive lysis buffer (Promega). Luciferase activity was detected using the Dual-Luciferase^® ^reporter assay system (Promega) according to the manufacture's instructions and the light output was measured with a luminometer (MONLIGHT^® ^1500, Analytical Luminescence Laboratory, San Diego, CA).

### Proliferation Assay

TERT-pSUPER and TERT-siSFRP1 cells (7.5 × 10^4 ^cells/well) were plated in 6-well dishes and the following day, the media was changed to control medium or Wnt3a medium containing 1 μCi/ml ^3^H-Thymidine (PerkinElmer, Waltham, MA). Twenty-four hours after the media change, cells were rinsed with 1× PBS and 10% trichloroacetic acid (TCA) was be added. The 10% TCA was removed and cells were washed again with 1× PBS. Next, cells were incubated in 0.1% SDS/0.1 N NaOH for 5 minutes at room temperature to lyse the cells. Lastly, 200 μl acetic acid and 800 μl of the cell lysate was added to 5 ml of scintillation and cpm values were collected using a scintillation counter (Tri-Carb 2900TR, PerkinElmer).

### Fluorescent Activated Cell Sorting (FACS)

For anoikis studies, 30 mm dishes were coated with 2 ml of 1% agarose/DMEM, which was allowed to polymerize creating a barrier that would prevent cellular attachment, and TERT-pSUPER or TERT-siSFRP1 cells (1.5 × 10^5 ^cells/well) were seeded in 2 ml of growth medium. After 24 hours, the media was collected and cells were pelleted by centrifugation. The pellet was resuspended in ice-cold 1× PBS, transferred into a round bottom 12 × 75 mm plastic culture tube (VWR international, NJ, USA), and incubated with 1 μg/ml propidium iodine (Invitrogen) in the dark for 15 minutes at room temperature to stain the dead cells. The ratio of dead cells/live cells was determined by flow cytometry (BD FACS Calibur, BD Bioscience, San Jose, CA).

For cell surface marker analysis, cells were washed once with PBS and then harvested with 0.05% trypsin/0.025% EDTA. Detached cells were washed with PBS containing 1% FBS and 1% penicillin/streptomycin (wash buffer), and resuspended in the wash buffer (10^6 ^cells/100 μl). Combinations of fluorochrome-conjugated monoclonal antibodies obtained from BD Biosciences against human CD44 (FITC; cat. #55478) and CD24 (PE; cat. #555428) and incubated at 4°C in the dark for 30 min. The labeled cells were washed in wash buffer and immediately analyzed by flow cytometry (BD FACS Calibur).

### Migration and Invasion Assays

For the scratch-wound assay, TERT-pSUPER and TERT-siSFRP1 cells were plated in 30 mm dishes, allowed to reach 100% confluence, and a pipette tip was utilized to generate a wound (scratch) down the center of the plate. Images of the cells capable of migrating across the scratch 8 hours after the wound were captured with a Nikon Eclipse TE2000-U using Metaview™ software (Universal Imaging Corporation). For chamber assays, TERT-pSUPER and TERT-siSFRP1 cells (5 × 10^5 ^cells/well) were seeded in serum free media in either BD BioCoat control chambers or Matrigel™ invasion chambers (BD Biosciences) above media containing 10% FBS. After a 22 hour incubation, chambers were removed and cells were fixed for 10 min in 10% formalin, stained for 10 min with 10% Crystal Violet, and rinsed 3× with dH_2_0. Non-migrating/invading cells were removed from the upper surface of the membrane by scrubbing the insert with a cotton tipped swab moistened with 1× PBS. The insert was removed from the chamber with a scalpel, placed on a microscope slide. Images were captured with an Olympic BX41 light microscope using SPOTSOFTWARE (Diagnostic Instruments, Inc, Sterling Hights, MI).

### Microarray Analysis

Agilent 4X44K human microarrays were performed by the Genomics Core Facility at University of North Carolina at Chapel Hill according to the manufacturers Amplification protocol. Four harvests at 60–80% confluence of the parent 76 N TERT cells were pooled and labeled with Cy3, while separate harvests (n = 4) of the SFRP1 knockdown cells were labeled in Cy5. Agilent feature extraction, loess normalization, and filtering of all spots with signal < 10 dpi was performed prior to supervised analysis. One class Significance Analysis of Microarrays [68] was used to identify genes that were significantly altered in knockdowns relative to parents with a False Detection Rate (FDR) < 5%.

### Statistical Analysis

Results were analyzed using a student's t-test or two-factor analysis of variance (ANOVA). Post-hoc tests, where appropriate, were performed by Bonferroni's t test, where the mean squared error term in the ANOVA table was used as the point estimate of the pooled variance (Prizm, GraphPad Software, Inc, San Diego, CA).

## Competing interests

The authors do not have any financial or personal relationships with other people or organizations that could inappropriately influence the work described in this manuscript.

## Authors' contributions

KG drafted the manuscript and performed all of the described experiments with the exception of the anoikis studies and the microarray analysis. MT performed the microarray analysis and edited the manuscript. JH performed the anoikis studies. SS participated in the study design and edited the manuscript.

## Supplementary Material

Additional File 1 **List of 130 genes validated differential expression between TERT-pSUPER and TERT-siSFRP1 cells**. This is a table which describes the probe ID, gene symbol, gene name, score, fold change, and standard deviation of the genes found to be differentially expressed according to microarray analysis. Click here for file

Additional File 2 **Primers used for real-time PCR analysis**. A list of the forward and reverse primers utilized in the real-time PCR assays described in the manuscript. Click here for file

## References

[B1] Hanahan D, Weinberg RA (2000). The hallmarks of cancer. Cell.

[B2] Polakis P (2000). Wnt signaling and cancer. Genes & development.

[B3] Brennan KR, Brown AM (2004). Wnt proteins in mammary development and cancer. Journal of mammary gland biology and neoplasia.

[B4] Karim R, Tse G, Putti T, Scolyer R, Lee S (2004). The significance of the Wnt pathway in the pathology of human cancers. Pathology.

[B5] Brown AM (2001). Wnt signaling in breast cancer: have we come full circle?. Breast Cancer Res.

[B6] Finch PW, He X, Kelley MJ, Uren A, Schaudies RP, Popescu NC, Rudikoff S, Aaronson SA, Varmus HE, Rubin JS (1997). Purification and molecular cloning of a secreted, Frizzled-related antagonist of Wnt action. Proceedings of the National Academy of Sciences of the United States of America.

[B7] Bafico A, Gazit A, Pramila T, Finch PW, Yaniv A, Aaronson SA (1999). Interaction of frizzled related protein (FRP) with Wnt ligands and the frizzled receptor suggests alternative mechanisms for FRP inhibition of Wnt signaling. The Journal of biological chemistry.

[B8] Zhou Z, Wang J, Han X, Zhou J, Linder S (1998). Up-regulation of human secreted frizzled homolog in apoptosis and its down-regulation in breast tumors. Int J Cancer.

[B9] Wong SC, Lo SF, Lee KC, Yam JW, Chan JK, Wendy Hsiao WL (2002). Expression of frizzled-related protein and Wnt-signalling molecules in invasive human breast tumours. J Pathol.

[B10] Klopocki E, Kristiansen G, Wild PJ, Klaman I, Castanos-Velez E, Singer G, Stohr R, Simon R, Sauter G, Leibiger H, Essers L, Weber B, Hermann K, Rosenthal A, Hartmann A, Dahl E (2004). Loss of SFRP1 is associated with breast cancer progression and poor prognosis in early stage tumors. International journal of oncology.

[B11] Ko J, Ryu KS, Lee YH, Na DS, Kim YS, Oh YM, Kim IS, Kim JW (2002). Human secreted frizzled-related protein is down-regulated and induces apoptosis in human cervical cancer. Experimental cell research.

[B12] Qi J, Zhu YQ, Luo J, Tao WH (2006). Hypermethylation and expression regulation of secreted frizzled-related protein genes in colorectal tumor. World J Gastroenterol.

[B13] Neuhausen A, Florl AR, Grimm MO, Schulz WA (2006). DNA methylation alterations in urothelial carcinoma. Cancer biology & therapy.

[B14] Marsit CJ, McClean MD, Furniss CS, Kelsey KT (2006). Epigenetic inactivation of the SFRP genes is associated with drinking, smoking and HPV in head and neck squamous cell carcinoma. Int J Cancer.

[B15] Liu TH, Raval A, Chen SS, Matkovic JJ, Byrd JC, Plass C (2006). CpG island methylation and expression of the secreted frizzled-related protein gene family in chronic lymphocytic leukemia. Cancer research.

[B16] Fukui T, Kondo M, Ito G, Maeda O, Sato N, Yoshioka H, Yokoi K, Ueda Y, Shimokata K, Sekido Y (2005). Transcriptional silencing of secreted frizzled related protein 1 (SFRP 1) by promoter hypermethylation in non-small-cell lung cancer. Oncogene.

[B17] Chang Q, Pang JC, Li KK, Poon WS, Zhou L, Ng HK (2005). Promoter hypermethylation profile of RASSF1A, FHIT, and sFRP1 in intracranial primitive neuroectodermal tumors. Hum Pathol.

[B18] Takada T, Yagi Y, Maekita T, Imura M, Nakagawa S, Tsao SW, Miyamoto K, Yoshino O, Yasugi T, Taketani Y, Ushijima T (2004). Methylation-associated silencing of the Wnt antagonist SFRP1 gene in human ovarian cancers. Cancer Sci.

[B19] Lee AY, He B, You L, Dadfarmay S, Xu Z, Mazieres J, Mikami I, McCormick F, Jablons DM (2004). Expression of the secreted frizzled-related protein gene family is downregulated in human mesothelioma. Oncogene.

[B20] Alves da Costa C, Mattson MP, Ancolio K, Checler F (2003). The C-terminal fragment of presenilin 2 triggers p53-mediated staurosporine-induced apoptosis, a function independent of the presenilinase-derived N-terminal counterpart. The Journal of biological chemistry.

[B21] Ugolini F, Charafe-Jauffret E, Bardou VJ, Geneix J, Adelaide J, Labat-Moleur F, Penault-Llorca F, Longy M, Jacquemier J, Birnbaum D, Pébusque MJ (2001). WNT pathway and mammary carcinogenesis: loss of expression of candidate tumor suppressor gene SFRP1 in most invasive carcinomas except of the medullary type. Oncogene.

[B22] Lo PK, Mehrotra J, D'Costa A, Fackler MJ, Garrett-Mayer E, Argani P, Sukumar S (2006). Epigenetic Suppression of Secreted Frizzled Related Protein 1 (SFRP1) Expression in Human Breast Cancer. Cancer biology & therapy.

[B23] Veeck J, Niederacher D, An H, Klopocki E, Wiesmann F, Betz B, Galm O, Camara O, Durst M, Kristiansen G, Huszka C, Knüchel R, Dahl E (2006). Aberrant methylation of the Wnt antagonist SFRP1 in breast cancer is associated with unfavourable prognosis. Oncogene.

[B24] Veeman MT, Slusarski DC, Kaykas A, Louie SH, Moon RT (2003). Zebrafish prickle, a modulator of noncanonical Wnt/Fz signaling, regulates gastrulation movements. Curr Biol.

[B25] Gilles C, Polette M, Zahm JM, Tournier JM, Volders L, Foidart JM, Birembaut P (1999). Vimentin contributes to human mammary epithelial cell migration. Journal of cell science.

[B26] Comijn J, Berx G, Vermassen P, Verschueren K, van Grunsven L, Bruyneel E, Mareel M, Huylebroeck D, van Roy F (2001). The two-handed E box binding zinc finger protein SIP1 downregulates E-cadherin and induces invasion. Molecular cell.

[B27] Eger A, Aigner K, Sonderegger S, Dampier B, Oehler S, Schreiber M, Berx G, Cano A, Beug H, Foisner R (2005). DeltaEF1 is a transcriptional repressor of E-cadherin and regulates epithelial plasticity in breast cancer cells. Oncogene.

[B28] Shirakihara T, Saitoh M, Miyazono K (2007). Differential regulation of epithelial and mesenchymal markers by deltaEF1 proteins in epithelial mesenchymal transition induced by TGF-beta. Molecular biology of the cell.

[B29] Aigner K, Dampier B, Descovich L, Mikula M, Sultan A, Schreiber M, Mikulits W, Brabletz T, Strand D, Obrist P, Sommergruber W, Schweifer N, Wernitznig A, Beug H, Foisner R, Eger A (2007). The transcription factor ZEB1 (deltaEF1) promotes tumour cell dedifferentiation by repressing master regulators of epithelial polarity. Oncogene.

[B30] Frisch SM, Francis H (1994). Disruption of epithelial cell-matrix interactions induces apoptosis. J Cell Biol.

[B31] Frisch SM, Screaton RA (2001). Anoikis mechanisms. Curr Opin Cell Biol.

[B32] Sheridan C, Kishimoto H, Fuchs RK, Mehrotra S, Bhat-Nakshatri P, Turner CH, Goulet R, Badve S, Nakshatri H (2006). CD44+/CD24- breast cancer cells exhibit enhanced invasive properties: an early step necessary for metastasis. Breast Cancer Res.

[B33] Mani SA, Guo W, Liao MJ, Eaton EN, Ayyanan A, Zhou AY, Brooks M, Reinhard F, Zhang CC, Shipitsin M, Campbell LL, Polyak K, Brisken C, Yang J, Weinberg RA (2008). The epithelial-mesenchymal transition generates cells with properties of stem cells. Cell.

[B34] Al-Hajj M, Wicha MS, Benito-Hernandez A, Morrison SJ, Clarke MF (2003). Prospective identification of tumorigenic breast cancer cells. Proceedings of the National Academy of Sciences of the United States of America.

[B35] Toledo F, Wahl GM (2006). Regulating the p53 pathway: in vitro hypotheses, in vivo veritas. Nature reviews.

[B36] Huff T, Muller CS, Otto AM, Netzker R, Hannappel E (2001). beta-Thymosins, small acidic peptides with multiple functions. The international journal of biochemistry & cell biology.

[B37] Smart N, Rossdeutsch A, Riley PR (2007). Thymosin beta4 and angiogenesis: modes of action and therapeutic potential. Angiogenesis.

[B38] Berube NG, Mangelsdorf M, Jagla M, Vanderluit J, Garrick D, Gibbons RJ, Higgs DR, Slack RS, Picketts DJ (2005). The chromatin-remodeling protein ATRX is critical for neuronal survival during corticogenesis. The Journal of clinical investigation.

[B39] Binger T, Stich S, Andreas K, Kaps C, Sezer O, Notter M, Sittinger M, Ringe J (2009). Migration potential and gene expression profile of human mesenchymal stem cells induced by CCL25. Experimental cell research.

[B40] Lee SF, Shah S, Li H, Yu C, Han W, Yu G (2002). Mammalian APH-1 interacts with presenilin and nicastrin and is required for intramembrane proteolysis of amyloid-beta precursor protein and Notch. The Journal of biological chemistry.

[B41] Wu F, Stutzman A, Mo YY (2007). Notch signaling and its role in breast cancer. Front Biosci.

[B42] Mohinta S, Wu H, Chaurasia P, Watabe K (2007). Wnt pathway and breast cancer. Front Biosci.

[B43] Nakopoulou L, Mylona E, Papadaki I, Kavantzas N, Giannopoulou I, Markaki S, Keramopoulos A (2006). Study of phospho-beta-catenin subcellular distribution in invasive breast carcinomas in relation to their phenotype and the clinical outcome. Mod Pathol.

[B44] Shulewitz M, Soloviev I, Wu T, Koeppen H, Polakis P, Sakanaka C (2006). Repressor roles for TCF-4 and Sfrp1 in Wnt signaling in breast cancer. Oncogene.

[B45] Sherr CJ (1995). D-type cyclins. Trends Biochem Sci.

[B46] Bartkova J, Lukas J, Muller H, Lutzhoft D, Strauss M, Bartek J (1994). Cyclin D1 protein expression and function in human breast cancer. Int J Cancer.

[B47] Kang Y, Massague J (2004). Epithelial-mesenchymal transitions: twist in development and metastasis. Cell.

[B48] Toyoda E, Doi R, Koizumi M, Kami K, Ito D, Mori T, Fujimoto K, Nakajima S, Wada M, Imamura M (2005). Analysis of E-, N-cadherin, alpha-, beta-, and gamma-catenin expression in human pancreatic carcinoma cell lines. Pancreas.

[B49] Bindels S, Mestdagt M, Vandewalle C, Jacobs N, Volders L, Noel A, van Roy F, Berx G, Foidart JM, Gilles C (2006). Regulation of vimentin by SIP1 in human epithelial breast tumor cells. Oncogene.

[B50] Gilles C, Polette M, Mestdagt M, Nawrocki-Raby B, Ruggeri P, Birembaut P, Foidart JM (2003). Transactivation of vimentin by beta-catenin in human breast cancer cells. Cancer research.

[B51] Hartwell LH, Kastan MB (1994). Cell cycle control and cancer. Science.

[B52] Seol MB, Bong JJ, Baik M (2005). Expression profiles of apoptosis genes in mammary epithelial cells. Mol Cells.

[B53] Wieczorek M, Paczkowska A, Guzenda P, Majorek M, Bednarek AK, Lamparska-Przybysz M (2007). Silencing of Wnt-1 by siRNA induces apoptosis of MCF-7 human breast cancer cells. Cancer biology & therapy.

[B54] He B, You L, Uematsu K, Xu Z, Lee AY, Matsangou M, McCormick F, Jablons DM (2004). A monoclonal antibody against Wnt-1 induces apoptosis in human cancer cells. Neoplasia.

[B55] Feng Q, Li P, Leung PC, Auersperg N (2004). Caspase-1zeta, a new splice variant of the caspase-1 gene. Genomics.

[B56] Feng Q, Li P, Salamanca C, Huntsman D, Leung PC, Auersperg N (2005). Caspase-1alpha is down-regulated in human ovarian cancer cells and the overexpression of caspase-1alpha induces apoptosis. Cancer research.

[B57] Komiya Y, Habas R (2008). Wnt signal transduction pathways. Organogenesis.

[B58] Nolan ME, Aranda V, Lee S, Lakshmi B, Basu S, Allred DC, Muthuswamy SK (2008). The polarity protein Par6 induces cell proliferation and is overexpressed in breast cancer. Cancer research.

[B59] Huang HC, Hu CH, Tang MC, Wang WS, Chen PM, Su Y (2007). Thymosin beta4 triggers an epithelial-mesenchymal transition in colorectal carcinoma by upregulating integrin-linked kinase. Oncogene.

[B60] Kobayashi T, Wang T, Maezawa M, Kobayashi M, Ohnishi S, Hatanaka K, Hige S, Shimizu Y, Kato M, Asaka M, Tanaka J, Imamura M, Hasegawa K, Tanaka Y, Brachmann RK (2006). Overexpression of the oncoprotein prothymosin alpha triggers a p53 response that involves p53 acetylation. Cancer research.

[B61] Wang WS, Chen PM, Hsiao HL, Wang HS, Liang WY, Su Y (2004). Overexpression of the thymosin beta-4 gene is associated with increased invasion of SW480 colon carcinoma cells and the distant metastasis of human colorectal carcinoma. Oncogene.

[B62] Ayyanan A, Civenni G, Ciarloni L, Morel C, Mueller N, Lefort K, Mandinova A, Raffoul W, Fiche M, Dotto GP, Brisken C (2006). Increased Wnt signaling triggers oncogenic conversion of human breast epithelial cells by a Notch-dependent mechanism. Proceedings of the National Academy of Sciences of the United States of America.

[B63] Collu GM, Brennan K (2007). Cooperation between Wnt and Notch signalling in human breast cancer. Breast Cancer Res.

[B64] Yogosawa S, Miyauchi Y, Honda R, Tanaka H, Yasuda H (2003). Mammalian Numb is a target protein of Mdm2, ubiquitin ligase. Biochemical and biophysical research communications.

[B65] Farnie G, Clarke RB (2007). Mammary stem cells and breast cancer – role of Notch signalling. Stem cell reviews.

[B66] Zang S, Ji C, Qu X, Dong X, Ma D, Ye J, Ma R, Dai J, Guo D (2007). A study on Notch signaling in human breast cancer. Neoplasma.

[B67] Chomczynski P, Sacchi N (1987). Single-step method of RNA isolation by acid guanidinium thiocyanate-phenol-chloroform extraction. Anal Biochem.

[B68] Tusher VG, Tibshirani R, Chu G (2001). Significance analysis of microarrays applied to the ionizing radiation response. Proceedings of the National Academy of Sciences of the United States of America.

