# Supplementary material for: Down-regulation of sfrp1 in a mammary epithelial cell line promotes the development of a cd44high/cd24low population which is invasive and resistant to anoikis
Source: Cancer Cell Int. 2009 May 7;9:11. doi: 10.1186/1475-2867-9-11 (PMC2687411; doi:10.1186/1475-2867-9-11)
Supplement: Additional File 1 — List of 130 genes validated differential expression between TERT-pSUPER and TERT-siSFRP1 cells. This is a table which describes the probe ID, gene symbol, gene name, score, fold change, and standard deviation of the genes found to be differentially expressed according to microarray analysis. [file 1475-2867-9-11-S1.pdf]

Table1. List of 130 genes with validated differential expression between TERT-pSUPER and TERT-siSFRP1 cells*.

Probe ID	Gene Symbol	Gene Name	Score (d)	Fold Change	SD
positively regulated genes					
A_23_P66011	NECAB2	EF hand calcium binding protein 2	2.859	3.588	1.555
A_23_P155796	GNRHR	gonadotropin-releasing hormone receptor	2.832	2.411	0.213
A_24_P160225	CXorf36	chromosome X open reading frame 36	2.731	2.420	0.278
A_32_P60013	C21orf135	chromosome 21 open reading frame 135	2.660	3.511	2.497
A_23_P416011	SPAG11B	sperm associated antigen 11B	2.617	3.041	0.962
A_32_P23113	FLNB	filamin B, beta (actin binding protein 278)	2.365	2.732	0.845
A_24_P817490			2.311	2.273	0.424
A_24_P934592			2.270	3.432	2.375
A_23_P424126	ZNF354C	zinc finger protein 354C	2.204	2.142	0.337
A_24_P331998	DOCK8	dedicator of cytokinesis 8	2.145	2.457	0.757
A_23_P47616	FOLH1	folate hydrolase (prostate-specific membrane antigen) 1	2.116	2.231	0.514
A_23_P78840	KIR2DS4	Killer cell immunoglobulin-like receptor, two domains, short cytoplasmic tail, 4	2.101	2.037	0.286
A_24_P932875	UNQ6490	similar to YPLR6490	2.077	2.039	0.305
A_24_P185368	ZSCAN4	zinc finger and SCAN domain containing 4	2.049	1.934	0.221
A_24_P24053			2.019	2.134	0.473
A_23_P35309	TAF5L	TAF5-like RNA polymerase II, p300/CBP-associated factor (PCAF)-associated factor, 65kDa	1.999	2.238	0.677
A_32_P70045			1.991	1.999	0.298
A_32_P93894			1.966	1.957	0.308
A_23_P214935	VNN3	vanin 3	1.963	2.066	0.434
A_23_P208706	BAX	BCL2-associated X protein	1.924	1.889	0.243
A_24_P942492	PALM2	paralemmin 2	1.923	1.931	0.297
A_23_P217570	CAPN6	calpain 6	1.865	1.895	0.307
A_24_P76512			1.854	1.853	0.230
A_32_P74771			1.843	2.111	0.553
A_32_P161667			1.819	2.036	0.497
A_24_P105891	DZIP1L	DAZ interacting protein 1-like	1.800	2.033	0.497
A_23_P34452	LOR	loricrin	1.795	1.711	0.105
A_24_P925901			1.793	1.960	0.452
A_23_P346311	BAX	BCL2-associated X protein	1.784	1.808	0.234
A_24_P281403			1.782	1.695	0.094
A_24_P933965			1.778	1.686	0.603
A_32_P131143	CECR4	cat eye syndrome chromosome region, candidate 4	1.773	1.898	0.391
A_32_P126214			1.764	1.915	0.393
A_23_P312565			1.763	2.072	0.607
A_24_P649507			1.750	2.128	0.769
A_32_P81334	LARP4	La ribonucleoprotein domain family, member 4	1.749	1.901	0.381
A_24_P401842	HHIP	hedgehog interacting protein	1.742	1.722	0.152
A_32_P60561	RELN	reelin	1.657	1.766	0.254
A_32_P145502	IGFBP3	insulin-like growth factor binding protein 3	1.657	1.595	0.449
A_24_P333993	OR2W3	olfactory receptor, family 2, subfamily W, member 3	1.646	1.620	0.082
A_23_P126613	AQP10	aquaporin 10	1.599	1.617	0.105
A_23_P332190	IRF8	interferon regulatory factor 8	1.596	1.654	0.156
A_23_P55828	CCL25	chemokine (C-C motif) ligand 25	1.595	1.902	0.570
A_32_P3602	USP3	ubiquitin specific peptidase 3	1.585	1.569	0.052
A_23_P33384	CIITA	class II, major histocompatibility complex, transactivator	1.579	1.739	0.299
A_23_P140490	BRUNOL6	bruno-like 6, RNA binding protein (Drosophila)	1.577	1.623	0.128
A_24_P388252	PPP3R1	protein phosphatase 3 (formerly 2B), regulatory subunit B, alpha isoform	1.569	1.840	0.435
A_24_P868334			1.549	1.599	0.112
A_24_P221235			1.548	1.797	0.426
A_23_P342869	FMN2	ormin 2	1.547	1.751	0.346
A_24_P263384			1.533	1.604	0.131
A_24_P702871	ZNF518A	zinc finger protein 518A	1.529	1.906	0.849
A_24_P51184			1.515	1.595	0.133
A_23_P321466	PEX11G	peroxisomal biogenesis factor 11 gamma	1.505	1.730	0.888
A_23_P5512			1.505	1.785	0.445
A_32_P17672			1.480	1.583	0.605
A_32_P34844			1.468	1.477	0.454
A_32_P199292			1.462	1.589	0.156
A_23_P52749	ADAMTS15	ADAM metallopeptidase with thrombospondin type 1 motif, 15	1.455	1.745	0.439
A_23_P348304	ADAMTSL1	ADAMTS-like 1	1.455	1.917	0.599
A_32_P117636	WWOX	WW domain containing oxidoreductase	1.446	1.721	0.363
A_32_P63069			1.446	1.549	0.109
A_32_P115438			1.430	1.418	0.334
A_24_P16606			1.404	1.788	0.536
A_24_P135406	KCTD9	potassium channel tetramerisation domain containing 9	1.403	1.462	0.009
A_24_P851254			1.401	2.125	1.145
positively regulated genes (cont.)					
A_24_P918907			1.394	1.502	0.387
A_23_P93169	GLULD1	glutamate-ammonia ligase (glutamine synthetase) domain containing 1	1.387	1.848	0.784
A_23_P104445	PIK3AP1	phosphoinositide-3-kinase adaptor protein 1	1.379	1.396	0.311
A_32_P6841			1.368	1.542	0.152
A_24_P932736			1.359	1.701	0.631
A_23_P210419	DLGAP4	discs, large (Drosophila) homolog-associated protein 4	1.353	1.552	0.188
A_24_P223163	NAF1	nuclear assembly factor 1 homolog (S. cerevisiae)	1.350	1.628	0.309
A_23_P309545	MDM2	Mdm2 p53 binding protein homolog (mouse)	1.345	1.485	0.080
A_24_P942036			1.338	1.362	0.283
A_32_P31827			1.337	1.594	0.262
A_23_P32078	SLC28A3	solute carrier family 28 (sodium-coupled nucleoside transporter), member 3	1.322	1.561	0.230
A_32_P752261			1.320	1.529	0.483
A_32_P26738	KIAA1143	KIAA1143	1.283	1.575	0.256
A_23_P254061	LUZPP1	leucine zipper protein pseudogene 1	1.275	1.524	0.190
A_24_P269895	HNRNPA3	heterogeneous nuclear ribonucleoprotein A3	1.273	1.478	0.118
A_23_P153286	ZNF234	zinc finger protein 234	1.272	1.520	0.182
A_24_P796274			1.271	1.616	0.341
A_24_P290927	UBAP2L	ubiquitin associated protein 2-like	1.260	1.748	0.742
A_32_P82189	FAM161A	family with sequence similarity 161, member A	1.260	1.565	0.263
A_24_P754185			1.248	1.664	0.488
A_23_P200772	ZNF644	zinc finger protein 644	1.247	1.561	0.279
A_23_P203150	TMPRSS13	transmembrane protease, serine 13	1.247	1.571	0.318
A_32_P111394			1.241	1.651	0.455
A_23_P2258	CCDC62	coiled-coil domain containing 62	1.239	1.603	0.400
A_23_P12329	APH1A	anterior pharynx defective 1 homolog A (C. elegans)	1.239	1.444	0.084
A_32_P85676	STK32B	serine/threonine kinase 32B	1.227	1.821	0.628
A_24_P65597	RPS19	ribosomal protein S19	1.224	2.577	3.148
A_32_P481631			1.224	1.476	0.153
A_24_P325992	LIFR	leukemia inhibitory factor receptor alpha	1.222	1.590	0.348
A_23_P114185	TSPAN7	tetraspanin 7	1.221	1.551	0.298
A_24_P315306	ITPRIPL2	inositol 1,4,5-triphosphate receptor interacting protein-like 2	1.213	1.424	0.069
A_24_P918575			1.209	1.502	0.203
A_24_P374516	TMSB4X	thymosin beta 4, X-linked	1.204	1.457	0.132
A_23_P376239	PAPOLA	poly(A) polymerase alpha	1.197	1.683	0.626
A_23_P407203	ZEB2	zinc finger E-box binding homeobox 2	1.194	1.493	0.195
A_24_P283294	MRPS10	mitochondrial ribosomal protein S10	1.194	1.443	0.114
A_24_P144936	PKD1L2	polycystic kidney disease 1-like 2	1.193	1.530	0.259
A_32_P378035			1.179	1.478	0.195
A_24_P43959	FRMD4A	FERM domain containing 4A	1.175	1.520	0.280
A_23_P211773	ACAP2	ArfGAP with coiled-coil, ankyrin repeat and PH domains 2	1.173	1.532	0.308
A_23_P214897	AKAP12	A kinase (PRKA) anchor protein 12	1.171	1.762	0.775
A_24_P79040	CAPN12	calpain 12	1.170	1.583	0.453
A_23_P358857	ZNF644	zinc finger protein 644	1.164	1.529	0.303
A_23_P115192	FCRL4	Fc receptor-like 4	1.162	1.566	0.348
A_32_P198978			1.160	1.731	0.562
A_23_P360754	ADAMTS2	ADAM metallopeptidase with thrombospondin type 1 motif, 2	1.157	1.619	0.471
A_24_P419323			1.157	1.410	0.084
A_24_P579984			1.150	1.509	0.284
A_24_P84719			1.150	1.490	0.227
A_24_P128041	ATRX	ATRX	1.149	1.445	0.155
A_23_P401606	EDIL3	EGF-like repeats and discoidin I-like domains 3	1.146	1.469	0.209
A_23_P81484			1.145	1.419	0.107
A_24_P93967	FMR1	fragile X mental retardation 1	1.144	1.443	0.160
A_24_P100650	C9orf11	chromosome 9 open reading frame 11	1.143	1.458	0.192
A_32_P185361	DYRK1A	dual-specificity tyrosine-(Y)-phosphorylation regulated kinase 1A	1.140	1.428	0.128
A_24_P867702			1.136	1.679	0.595
A_24_P926450			1.129	1.528	0.330
A_24_P162485	ANK1	ankyrin 1, erythrocytic	1.128	1.321	0.282
A_24_P25252	ANKRD12	ankyrin repeat domain 12	1.128	1.515	0.293
A_23_P64529			1.123	1.474	0.243
A_24_P392265	EXOC5	exocyst complex component 5	1.121	1.477	0.238
A_32_P157228	AHCTF1	AT hook containing transcription factor 1	1.121	1.526	0.342
negatively regulated genes					
A_24_P852756	HLA-DQA2	major histocompatibility complex, class II, DQ alpha 2	-1.940	-2.190	0.640
A_32_P195515	IQSEC3	IQ motif and Sec7 domain 3	-1.917	-1.840	0.180

* This table was compiled by generating the largest possible gene list with a < 5% false discovery rate.
