# Supplementary material for: Down-regulation of sfrp1 in a mammary epithelial cell line promotes the development of a cd44high/cd24low population which is invasive and resistant to anoikis
Source: Cancer Cell Int. 2009 May 7;9:11. doi: 10.1186/1475-2867-9-11 (PMC2687411; doi:10.1186/1475-2867-9-11)
Supplement: Additional File 2 — Primers used for real-time PCR analysis. A list of the forward and reverse primers utilized in the real-time PCR assays described in the manuscript. [file 1475-2867-9-11-S2.pdf]

Table 2. Primers utilized for real-time PCR analysis

Amplicon	Forward Primer	Reverse Primer
SFRP1	5’-GCCCGAGATGCTTAAGTGTGACAA-3’	5’- ACTCGCTGGCACAGAGATGTTCAA-3’
Cyclin D1	5’-AACTACCTGGACCGCTTCCT -3'	5’- CCACTTGAGCTTGTTCACCA-3’
E-cadherin	5'-ACACCATCCTCAGCCAAGA-3'	5'-CGTAGGGAAACTCTCTCGGT-3'
vimentin	5'-AAAGTGTGGCTGCCAAGAAC-3'	5'-AGCCTCAGAGAGGTCAGCAA-3'
Snail	5'-GCTGCAGGACTCTAATCCAGA-3'	5'-ATCTCCGGAGGTGGGATC-3'
Slug	5'-CATGCCTGTCATACCACAAC-3'	5'-GGTGTCAGATGGAGGAGGG-3'
ZEB1	5'- GGGAGGAGCAGTGAAAGAGA-3'	5'-TTTCTTGCCCTTCCTTTCTG-3'
ZEB2	5'- AAGCCAGGGACAGATCAGC-3'	5'-CCACACTCTGTGCATTTGAACT-3'
GAPDH	5’-GAAGATGGTGATGGGATTTC-3’	5’-GAAGGTGAAGGTCGGAGT -3’
